# Neglected painless wounds in a child with congenital insensitivity to pain

**DOI:** 10.11604/pamj.2014.17.95.3940

**Published:** 2014-02-07

**Authors:** Ali Akhaddar, Mohamed Malih

**Affiliations:** 1 Department of Neurosurgery, Avicenne Military Hospital, Marrakech, Morocco; 2 University of Mohammed V Souissi, Rabat, Morocco; 3 Department of Pediatrics, Mohammed V Military Teaching Hospital, Rabat, Morocco

**Keywords:** Congenital insensitivity, self-mutilation, neuropathy

## Image in medicine

A 13-year-old boy was brought by his mother for neglected toes wounds. There was a history of self-mutilation from first years of life with absence of normal reaction to painful stimuli. He had scars from injuries and he had self-mutilation of the distal thumb of the second finger on the right hand (A) and the first toe was missing on the left foot (B). Neurologic examination revealed decreased tactile sensitivity. The child seemed to respond appropriately to thermal stimuli. However, there was no reaction to pain stimuli. It was noted that he had normal mental development. Hands and feet radiograph showed destruction and amputation of the second right finger (C) and the first left toe (D). Biologic data were normal. There was no consanguinity but one of his paternal uncles seems to have a similar medical history. The infected wounds were treated conservatively with antibiotics and local care. Congenital insensitivity to pain with anhidrosis is extremely rare entity also known as hereditary sensory and autonomic neuropathy type IV. This autosomal recessive disorder is characterized by the congenital lack of pain sensation, inability to sweat, episodes of recurrent hyperpyrexia, mental retardation, and self-mutilating behavior. Patients are subjected to repeated injuries which are often neglected. There is no specific treatment but patient training and parent education are keys to avoid further neglect and damage.

**Figure 1:**
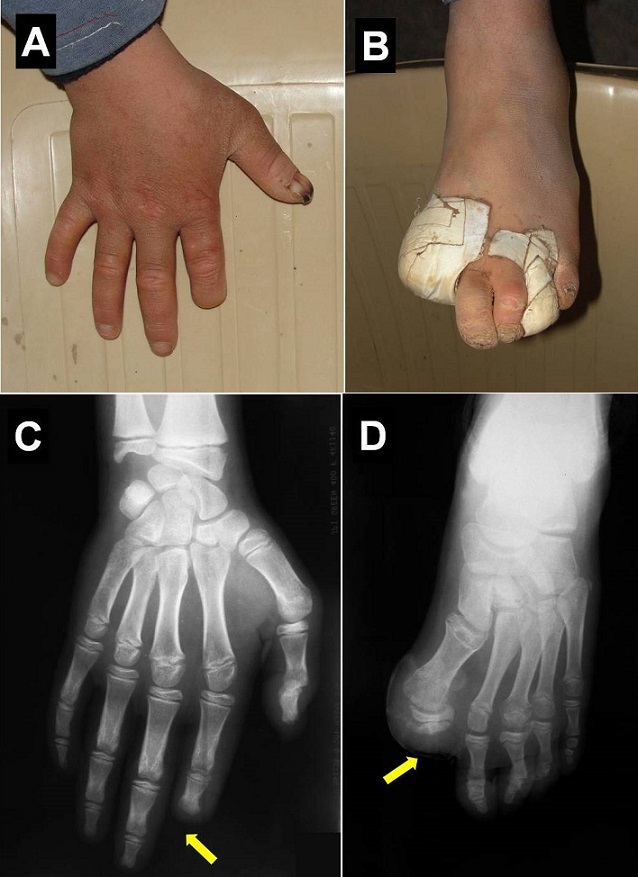
A) External photographs of right hand and left foot; B) showing self-mutilation of the distal phalanx of the second finger and the first toe was missing Hand (C) and foot (D) radiographs revealing destruction and amputation of the terminal part of the second right finger and the first left toe (arrows)

